# Supplementary material for: Haematopoietic stem cell gene therapy with IL‐1Ra rescues cognitive loss in mucopolysaccharidosis IIIA
Source: EMBO Mol Med. 2020 Feb 14;12(3):e11185. doi: 10.15252/emmm.201911185 (PMC7059006; doi:10.15252/emmm.201911185)
Supplement: Supplementary file 1 — Appendix [file EMMM-12-e11185-s001.pdf]

Haematopoietic stem cell gene therapy with IL-1Ra rescues cognitive loss in mucopolysaccharidosis IIIA

Helen Parker, Stuart M Ellison, Rebecca J Holley, Claire O'Leary, Aiyin Liao, Jalal Asadi, Emily Glover, Arunabha Ghosh, Simon Jones, Fiona L Wilkinson, David Brough, Emmanuel Pinteaux, Herve Boutin & Brian W Bigger

Appendix

Table of contents

Appendix Table S1, page 2
Appendix Table S2, page 2
Appendix Table S3, page 3
Appendix Table S4, page 3
Appendix Table S5, page 3
Appendix Table S6, page 4
Appendix Table S7, page 4

Appendix Table S1. List of exact P-values for graphs in Figure 1.

Figure	Panel	Comparison	*P*-value
1	B	Tnfa; WT vs. MPSIIIA	*P* <0.0001
1	B	Il1b; WT vs. MPSIIIA	*P* = 0.0004
1	B	Il1a; WT vs. MPSIIIA	*P* = 0.0168
1	B	Il6; WT vs. MPSIIIA	*P* = 0.0089
1	B	Il1rn; WT vs. MPSIIIA	*P* = 0.0002

Appendix Table S2. List of exact P-values for graphs in Figure 2.

Figure	Panel	Comparison	*P*-value
2	B	Within 2 hours; Saline vs. MPSIIIA GAG	*P* <0.0001
2	B	Within 6 hours; Saline vs. MPSIIIA GAG	*P* <0.0001
2	B	Within MPSIIIA GAG; 1 hour vs. 2 hours	*P* <0.0001
2	B	Within MPSIIIA GAG; 1 hour vs. 6 hours	*P* = 0.0178
2	B	Within MPSIIIA GAG; 2 hours vs. 6 hours	*P* = 0.0096
2	B	Within 1 hour; Saline vs. LPS	*P* <0.0001
2	B	Within 2 hours; Saline vs. LPS	*P* <0.0001
2	B	Within 6 hours; Saline vs. LPS	*P* <0.0001
2	D	PBS vs. MPSIIIA GAG	*P* <0.0001
2	D	PBS vs. LPS	*P* <0.0001
2	D	WT GAG vs. MPSIIIA GAG	*P* <0.0001
2	E	PBS vs. MPSIIIA	*P* = 0.0033
2	E	PBS vs. LPS	*P* <0.0001
2	F	Vehicle vs. MPSIIIA GAG+vehicle	*P* <0.0001
2	F	MPSIIIA GAG+vehicle vs. MPSIIIA GAG+CLI-095	*P* <0.0001
2	G	MPSIIIA GAG vs. MPSIIIA GAG+cABC	*P* = 0.0007
2	G	MPSIIIA GAG vs. MPSIIIA GAG+HepM	*P* <0.0001
2	G	MPSIIIA GAG vs. MPSIIIA GAG+HepM+cABC	*P* <0.0001
2	G	MPSIIIA GAG+cABC vs. MPSIIIA GAG+HepM	*P* <0.0001
2	G	MPSIIIA GAG+cABC vs. MPSIIIA GAG+HepM+cABC	*P* <0.0001
2	G	MPSIIIA GAG+HepM vs. MPSIIIA GAG+HepM+cABC	*P* = 0.0164

Appendix Table S3. List of exact P-values for graphs in Figure 3.

Figure	Panel	Comparison	*P*-value
3	C	Pycard; WT vs. MPSIIIA	*P* = 0.0241
3	C	Nlrp3; WT vs. MPSIIIA	*P* = 0.0283
3	C	Casp1; WT vs. MPSIIIA	*P* = 0.0131
3	C	Ctsb; WT vs. MPSIIIA	*P* = 0.0416
3	C	Casp4; WT vs. MPSIIIA	*P* = 0.0438
3	C	Gsdmd; WT vs. MPSIIIA	*P* = 0.0320
3	E	Within MPSIIIA GAG; Vehicle vs. ATP	*P* = 0.0002
3	E	Within LPS; Vehicle vs. ATP	*P* = 0.0492
3	F	Within MPSIIIA GAG; Vehicle vs. CHC	*P* = 0.0001
3	F	Within LPS; Vehicle vs. CHC	*P* = 0.0027
3	G	Within MPSIIIA GAG; Vehicle vs. rhAβ	*P* = 0.0052
3	G	Within LPS; Vehicle vs. rhAβ	*P* = 0.0062
3	H	Vehicle alone vs. LPS+vehicle	*P* = 0.0006
3	H	Vehicle alone vs. LPS+ATP	*P* = 0.0001
3	I	Vehicle alone vs. LPS+vehicle	*P* <0.0001
3	I	Vehicle alone vs. LPS+CHC	*P* <0.0001
3	J	Vehicle alone vs. LPS+vehicle	*P* <0.0001
3	J	Vehicle alone vs. LPS+ rhAβ	*P* <0.0001

Appendix Table S4. List of exact P-values for graphs in Figure 4.

Figure	Panel	Comparison	*P*-value
4	B	Within NTC; no treatment vs. 10 ng/ml IL-1β	*P* <0.0001
4	B	Within LV.GFP; no treatment vs. 10 ng/ml IL-1β	*P* <0.0001
4	B	Within 10 ng/ml IL-1β; NTC vs. LV.IL1RN	*P* <0.0001
4	B	Within 10 ng/ml IL-1β; LV.GFP vs. LV.IL1RN	*P* <0.0001

Appendix Table S5. List of exact P-values for graphs in Figure 5.

Figure	Panel	Comparison	*P*-value
5	C	WT vs. MPSIIIA	*P* = 0.0348
5	C	MPSIIIA vs. MPSIIIA+LV.IL1RN	*P* = 0.0062
5	C	MPSIIIA vs. MPSIIIAxIL-1R1$^{-/-}$	*P* = 0.0278
5	E	WT vs. MPSIIIA	*P* <0.0001
5	E	MPSIIIA vs. MPSIIIA+LV.IL1RN	*P* = 0.0002
5	E	MPSIIIA vs. MPSIIIAxIL-1R1$^{-/-}$	*P* <0.0001

Appendix Table S6. List of exact P-values for graphs in Figure 6.

Figure	Panel	Comparison	*P*-value
6	C	WT vs. MPSIIIA	*P* <0.0001
6	C	WT vs. MPSIIIA+LV.IL1RN	*P* = 0.0023
6	C	WT vs. MPSIIIAxIL-1R1$^{-/-}$	*P* = 0.0040
6	C	MPSIIIA vs. MPSIIIA+LV.IL1RN	*P* <0.0001
6	C	MPSIIIA vs. MPSIIIAxIL-1R1$^{-/-}$	*P* <0.0001
6	C	WTxIL-1R1$^{-/-}$ vs. MPSIIIAxIL-1R1$^{-/-}$	*P* <0.0001
6	D	WT vs. MPSIIIA	*P* <0.0001
6	D	WT vs. MPSIIIA+LV.IL1RN	*P* <0.0001
6	D	WT vs. MPSIIIAxIL-1R1$^{-/-}$	*P* <0.0001
6	D	MPSIIIA vs. MPSIIIA+LV.IL1RN	*P* <0.0001
6	D	MPSIIIA vs. MPSIIIAxIL-1R1$^{-/-}$	*P* <0.0001
6	D	WTxIL-1R1$^{-/-}$ vs. MPSIIIAxIL-1R1$^{-/-}$	*P* <0.0001
6	E	WT vs. MPSIIIA	*P* <0.0001
6	E	WT vs. MPSIIIA+LV.IL1RN	*P* <0.0001
6	E	WT vs. MPSIIIAxIL-1R1$^{-/-}$	*P* <0.0001
6	E	MPSIIIA vs. MPSIIIA+LV.IL1RN	*P* <0.0001
6	E	MPSIIIA vs. MPSIIIAxIL-1R1$^{-/-}$	*P* <0.0001
6	E	WTxIL-1R1$^{-/-}$ vs. MPSIIIAxIL-1R1$^{-/-}$	*P* <0.0001
6	F	WT vs. MPSIIIA	*P* <0.0001
6	F	WT vs. MPSIIIA+LV.IL1RN	*P* <0.0001
6	F	WT vs. MPSIIIAxIL-1R1$^{-/-}$	*P* <0.0001
6	F	MPSIIIA vs. MPSIIIA+LV.IL1RN	*P* <0.0001
6	F	MPSIIIA vs. MPSIIIAxIL-1R1$^{-/-}$	*P* <0.0001
6	F	MPSIIIA+LV.IL1RN vs. MPSIIIAxIL-1R1$^{-/-}$	*P* <0.0001
6	F	WTxIL-1R1$^{-/-}$ vs. MPSIIIAxIL-1R1$^{-/-}$	*P* <0.0001

Appendix Table S7. List of exact P-values for graphs in Expanded View Figures.

Figure	Panel	Comparison	*P*-value
EV1	B	UA2S-GlcNS6S; WT vs. MPSIIIA	*P* <0.0001
EV1	B	UA-GlcNS6S; WT vs. MPSIIIA	*P* = 0.0044
EV1	B	UA2S-GlcNS; WT vs. MPSIIIA	*P* <0.0001
EV1	B	UA-GlcNS; WT vs. MPSIIIA	*P* <0.0001
EV1	B	UA-GlcNAc6S; WT vs. MPSIIIA	*P* <0.0001
EV1	B	UA-GlcNAc; WT vs. MPSIIIA	*P* <0.0001
EV2		MPSIIIA GAG vs. MPSIIIA GAG+CHC	*P* <0.0001
EV2		MPSIIIA GAG+CHC vs. MPSIIIA GAG+CHC+VX-765	*P* = 0.0003
EV2		MPSIIIA GAG+CHC vs. MPSIIIA GAG+CHC+CA-074Me	*P* = 0.0001
EV2		MPSIIIA GAG+CHC vs. MPSIIIA GAG+CHC+CytD	*P* = 0.0004
EV2		MPSIIIA GAG+CHC vs. MPSIIIA GAG+CHC+KCl	*P* = 0.0003
EV3	B	WT vs. MPSIIIA	*P* = 0.0097
EV3	B	WT vs. WTxIL-1R1$^{-/-}$	*P* = 0.0314
EV3	B	MPSIIIA vs. MPSIIIA+LV.IL1RN	*P* = 0.0071
EV3	B	MPSIIIA+LV.IL1RN vs. MPSIIIAxIL-1R1$^{-/-}$	*P* = 0.0158
EV4	B	WT vs. MPSIIIA	*P* = 0.0003
EV4	B	WT vs. MPSIIIA+LV.IL1RN	*P* = 0.0078
EV4	B	WT vs. MPSIIIAxIL-1R1$^{-/-}$	*P* = 0.0348
EV4	B	WTxIL-1R1$^{-/-}$ vs. MPSIIIAxIL-1R1$^{-/-}$	*P* = 0.0008
