# Supplementary material for: Haematopoietic stem cell gene therapy with IL‐1Ra rescues cognitive loss in mucopolysaccharidosis IIIA
Source: EMBO Mol Med. 2020 Feb 14;12(3):e11185. doi: 10.15252/emmm.201911185 (PMC7059006; doi:10.15252/emmm.201911185)
Supplement: Supplementary file 2 — Expanded View Figures PDF [file EMMM-12-e11185-s002.pdf]

Expanded View Figures

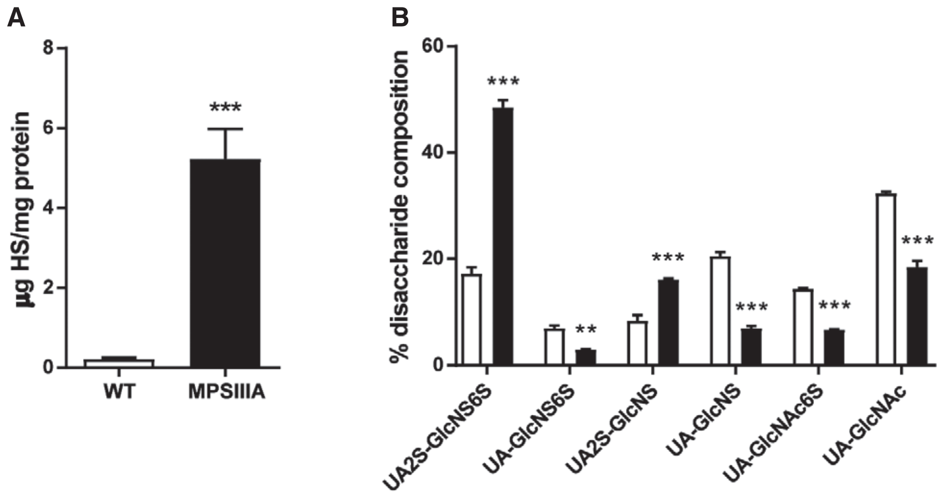

Figure EV1. Heparan sulphate analysis.

A Total relative amounts of HS. Amounts are expressed as μg HS per mg of liver protein and calculated from AMAC fluorescent reads compared to known amounts of HS standards (n = 3 GAG samples). Data are expressed as mean ± STDEV, and data for each cytokine were tested by unpaired *t*-test; WT versus MPSIIIA ***$P < 0.0001$.

B Compositional disaccharide analysis for HS from WT and MPSIIIA (n = 3 GAG samples). Data are expressed as mean ± STDEV and were tested by one-way ANOVA with Tukey's post-test; **$P < 0.01$, ***$P < 0.001$. Symbols above bars are versus WT. Exact *P*-values are indicated in Appendix Table S7. *NAc*, *N*-acetylated glucosamine; *NS*, N-sulphated glucosamine; *2S*, 2-*O*-sulphate group; *6S*, 6-*O*-sulphate group.

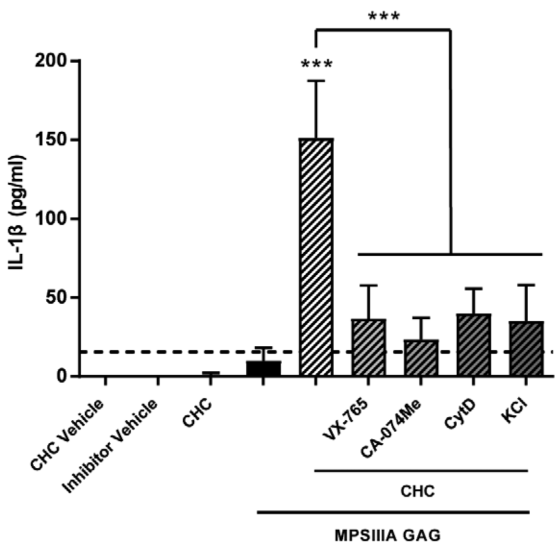

Figure EV2. MPSIIIA secondary storage substrates induce secretion of IL-1β via the NLRP3 inflammasome.

WT-mixed glia were incubated with cholesterol crystals in the presence or absence of caspase-1 inhibitor VX-765 (50 μg/ml), cytochalasin D (2 μM), KCl (130 mM) or cathepsin B inhibitor CA-074Me (10 μM). After the incubation, cell culture supernatants were analysed for IL-1β (n = 3 independent experiments each with three intra experimental replicates). Data are expressed as mean ± STDEV and were tested by one-way ANOVA with Tukey's post-test; ***$P < 0.001$. Symbols above bars are versus MPSIIIA GAG alone. Exact *P*-values are indicated in Appendix Table S7.

© 2020 The Authors

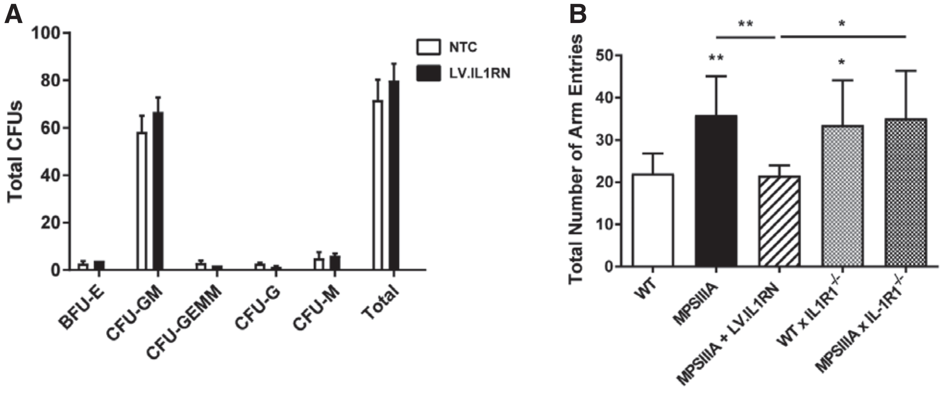

Figure EV3. Further evaluation of LV.IL1RN HSCT safety and efficacy.

A Colony-forming units from lineage-negative-enriched haematopoietic stem cells. Lineage-negative-enriched stem cells were either transduced with LV.CD11b.IL1RN or mock transduced and plated for 14 days in methylcellulose culture medium yielding committed haematopoietic progenitor colonies (n = 6CFU assays). Data are expressed as mean ± STDEV and were tested by one-way ANOVA with Tukey's post-test.

B Locomotor activity in the Y-maze. The total number of arm entries was assessed as an indicator of explorative behaviour (n = 10 mice per group). Data are expressed as mean ± STDEV and were tested by one-way ANOVA with Tukey's post-test; *$P < 0.05$, **$P < 0.01$. Symbols above bars are versus WT. Exact P-values are indicated in Appendix Table S7.

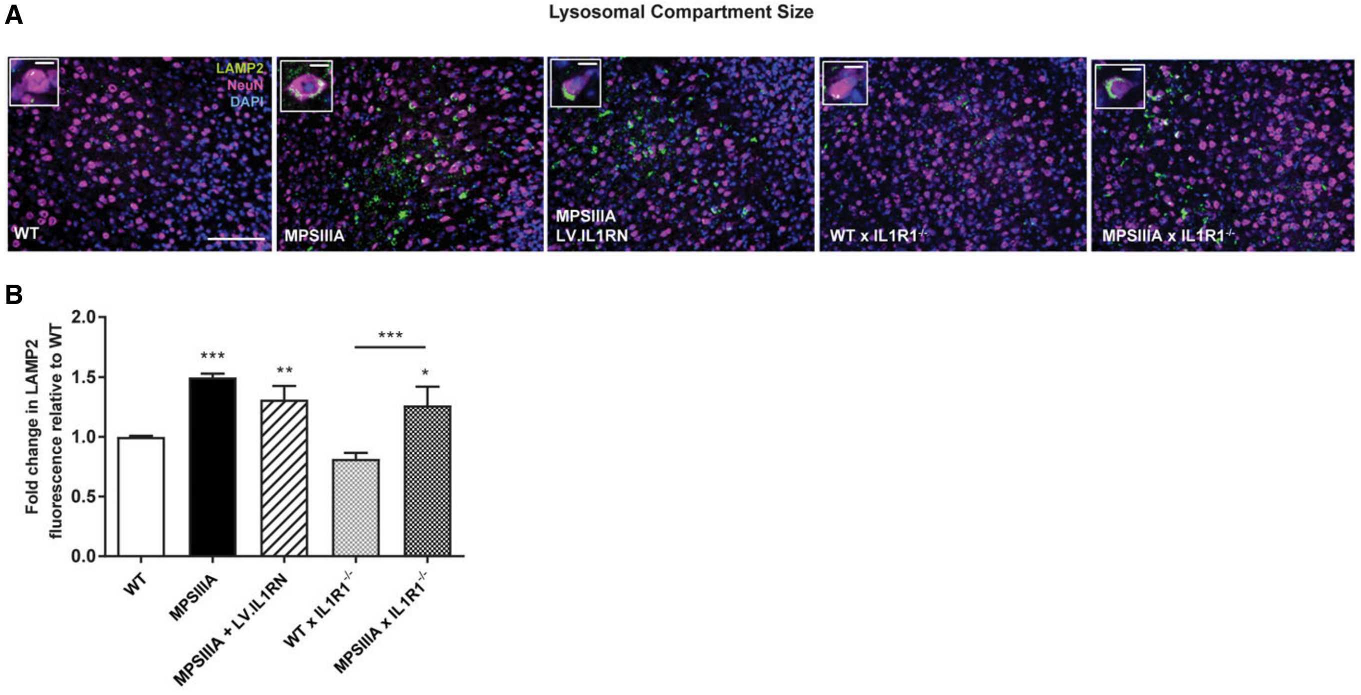

Figure EV4. IL-1 signalling inhibition in MPSIIIA mice has no effect on lysosomal storage.

A Representative images of the cerebral cortex (layers II-V) from control and treated mice stained with LAMP2 (lysosomal size, green)/NeuN (neuronal marker, magenta)/DAPI (nuclear, blue), 20×, scale bar: 50 µm. Inserts, 100×, scale bar: 10 µm.

B Quantification of fluorescence intensity using ImageJ software. Non-linear adjustments were made equally in fluorescent images to reduce background (n = 4 mice per group, average of three fields of view per mouse). Data are expressed as mean ± STDEV and were tested by one-way ANOVA with Tukey's post-test; *$P < 0.05$, **$P < 0.01$, ***$P < 0.001$. Symbols above bars are versus WT. Exact P-values are indicated in Appendix Table S7.

© 2020 The Authors
