# Supplementary material for: Haematopoietic stem cell gene therapy with IL‐1Ra rescues cognitive loss in mucopolysaccharidosis IIIA
Source: EMBO Mol Med. 2020 Feb 14;12(3):e11185. doi: 10.15252/emmm.201911185 (PMC7059006; doi:10.15252/emmm.201911185)
Supplement: Supplementary file 3 — Table EV1 [file EMMM-12-e11185-s003.docx]

**Extended View Table**

## Table EV1 Gene expression assays used to assess neuroinflammatory mediator expression

| Gene | Assay ID | Gene | | Assay ID |
| --- | --- | --- | --- | --- |
| *Tnfa* | Mm00443258_m1 | ***Ly96 (Md2)*** | Mm01227593_m1 | |
| *Il1b* | Mm00434228_m1 | ***Lbp*** | Mm00493139_m1 | |
| *Il1a* | Mm00439620_m1 | ***Nlrp3*** | Mm00840904_m1 | |
| *Il6* | Mm00446190_m1 | ***Pycard (Asc)*** | Mm00445747_g1 | |
| *Ccl2* | Mm00441242_m1 | ***Casp1*** | Mm00438023_m1 | |
| *Ccl3* | Mm00441259_g1 | ***Ctsb*** | Mm01310506_m1 | |
| *Il1rn* | Mm00446186_m1 | ***Gsdmd*** | Mm00509958_m1 | |
| *Tlr4* | Mm00445273_m1 | ***Casp4 (11)*** | Mm00432304_m1 | |
| *Cd14* | Mm00438094_g1 | ***Gapdh*** | Mm99999915_g1 | |
| *Cd44* | Mm01277161_m1 |  |  | |
