# Supplementary figures and images for: Haematopoietic stem cell gene therapy with IL‐1Ra rescues cognitive loss in mucopolysaccharidosis IIIA
Source: EMBO Mol Med. 2020 Feb 14;12(3):e11185. doi: 10.15252/emmm.201911185 (PMC7059006; doi:10.15252/emmm.201911185)

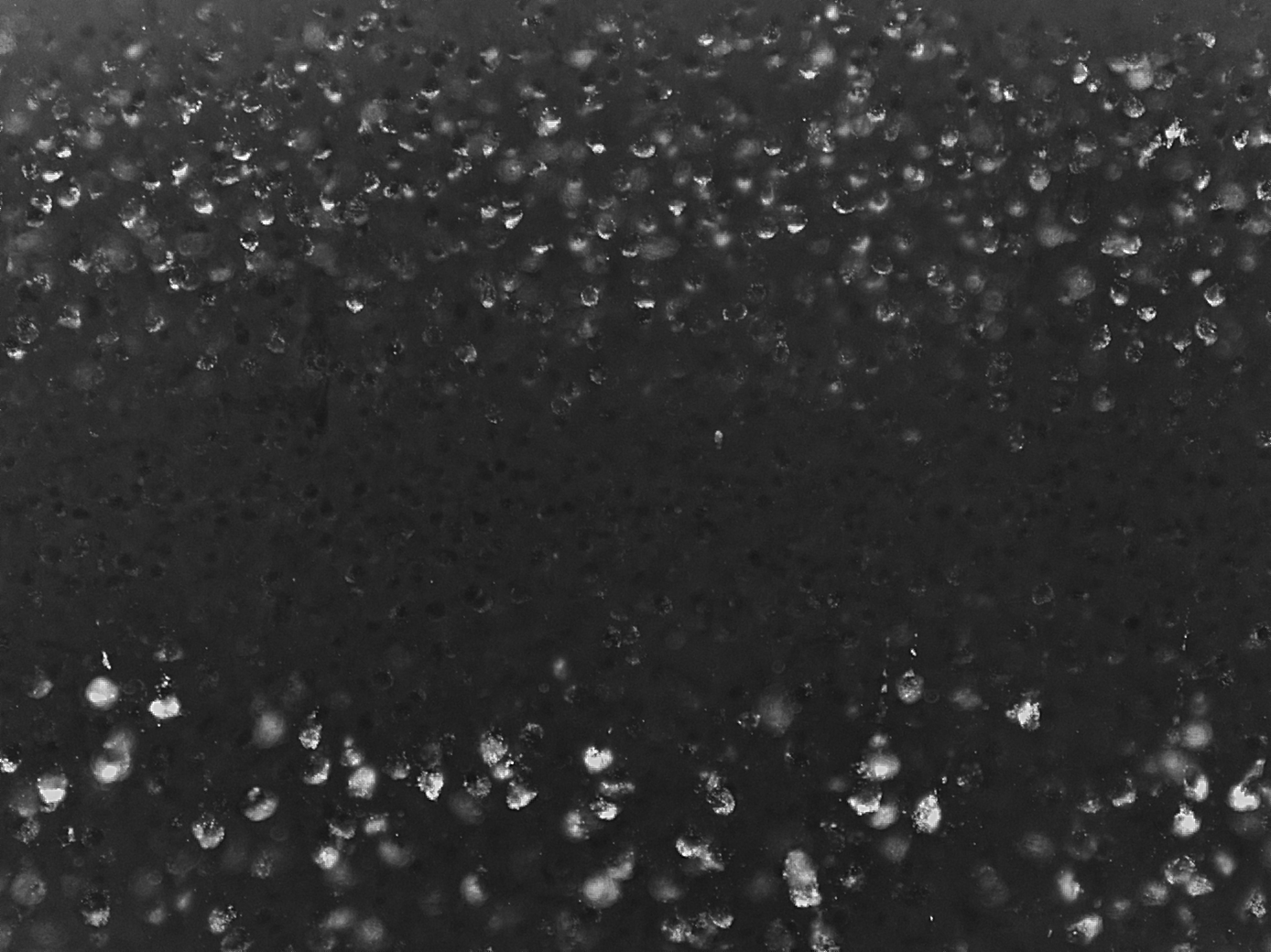

Supplement: Supplementary file 5 — Source Data for Figure 3 [file EMMM-12-e11185-s004.tif]
